# Supplementary material for: Are carbon emissions trading and green financial instruments synergistic? -Comprehensive quantitative research based on content analysis
Source: PLoS One. 2024 Mar 7;19(3):e0298601. doi: 10.1371/journal.pone.0298601 (PMC10919601; doi:10.1371/journal.pone.0298601)
Supplement: S1 Data — (ZIP) [file pone.0298601.s001.zip › Data/地级市Z_F工作报告.zip!/╜¡╬≈/╛░╡┬╒≥╩╨/└┤╫╘╣1⁄2╓┌║┼í╛┬φ┐╦╩2╛▌═°í┐_2008.docx]

各位代表：

　　现在，我代表市人民政府向大会作政府工作报告，请予审议，并请各位市政协委员和列席会议的同志提出意见。

　　一、2007年的工作回顾

　　过去的一年，在省委、省政府和市委的正确领导下，全市上下坚持以邓小平理论和“三个代表”重要思想为指导，以迎接十七大、学习十七大、贯彻十七大为主线，以科学发展观统领经济社会发展全局，全面落实市第九次党代会提出的“科学发展，和谐兴市，实现建设经济重镇、旅游都市、特色瓷都新跨越”的任务，以“3199工程”为抓手，重大项目建设取得新进展，卓有成效地实现了为新跨越打基础的目标，经济社会实现了又好又快发展。

　　2007年，预计全市实现生产总值270亿元，增长14.5%，三次产业结构调整为8.59：57.81：33.6 ，二产比重提高3.6个百分点；完成财政总收入20.03亿元，增长22.7%，其中一般预算收入11.9亿元，增长22.6%；城镇固定资产投资108亿元，增长21%；社会消费品零售总额80亿元，增长17%；城镇居民人均可支配收入12000元，增长20.5%；农民人均纯收入4463元，增长12.8%；人口自然增长率控制在7.75 ‰以内。

　　开放型经济取得重大突破。内外资快速增长，预计全年内资引进5000万以上的工业项目和亿元以上的综合类项目46个，实际到位资金48亿元，增长45.5%，完成省政府任务的240%；实际利用外资7800万美元，增长31.4%。汽车零配件产业基地、长虹机电产业基地、乐华、金意陶、特地和华润天然气等一批重大项目相继落户我市开工建设。园区规模以上企业达123家，完成基础设施投入2亿元，同比分别增长48.2%和22%。全年外贸出口达1.6亿美元，增长33.1%，机电、陶瓷和医药化工三大类产品出口均创历史新高。对外经济技术合作积极推进，海外市场进一步开拓。海关、检验检疫通关条件日益改善，通关效率不断提高。

　　工业经济快速增长。预计全年完成规模以上工业增加值95亿元，增长27.2%；销售收入240亿元，增长28%；利税总额10亿元，增长59.7%。园区发展势头强劲，预计园区完成规模以上工业增加值32亿元，占全市三分之一，比上年提高13.9个百分点。乐平工业园区被评为省级先进工业园区。国有企业改革稳步推进，华意压缩成功实施战略性重组。不良债权收购、处置工作取得重大进展，收购8个资产公司11个不良资产包，涉及债务企业400多户，化解债务32亿元，实现了国有资产保值增值，维护了社会和企业稳定。企业创新能力不断增强，实施技术创新项目35项。

　　陶瓷总量不断做大。预计全年实现陶瓷总产值42亿元，增长31%；陶瓷出口完成5030万美元，增长31%；陶瓷产业实现税收4000万元，创历史最好水平；新增规模以上陶瓷企业17户，产值亿元以上陶瓷企业3家。陶瓷工业园区基础设施日臻完善，4万平方米标准化厂房已交付使用；新入园项目43个，其中18个建成投产，初步形成了高技术陶瓷、日用陶瓷、艺术陶瓷、建筑卫生陶瓷和相关配套产业的聚集。2007景德镇国际陶瓷博览会取得丰硕成果，内贸成交6.17亿元，增长18.4%，外贸成交1亿多美元，增长22.7%。陶瓷科研基地建设不断加强，“国家陶瓷产品质量监督检验中心（江西）”通过评审和验收，国家日用及建筑陶瓷工程技术研究中心通过科技部专家组的现场验收评估。

　　农村经济稳步发展。预计全年完成农业总产值38.5亿元，增长6.1%。粮食连续四年保持增产，总产创历史新纪录，达到51.5万吨。社会主义新农村建设取得新进展，新农村建设完成投入1.25亿元。农业产业化水平进一步提升，乐平市荣获“江南菜乡”称号，乐平蔬菜集团成为全省10家销售过10亿元的龙头企业之一；浮梁县被评为国家级茶叶标准化示范县创建单位，茶叶总产值1.23亿元；昌江区培育发展省级农业产业化龙头企业10家，养殖业总产值2亿元。水利管理体制改革圆满完成，种子管理体制和畜牧兽医管理体制改革取得阶段性成果，林业产权制度主体改革基本完成。绿化造林工作扎实推进，2007年造林面积10.5万亩。“造地增粮富民工程”完成造地1560亩。农业综合开发投入2125万元。气象、水文防灾减灾能力进一步加强。劳务经济发展势头强劲，预计全年外出务工农民25.1万人，增长9.1% ，促进农民人均增收185元。

　　旅游产业不断提升。预计全年接待国内游客760万人次，增长15%，境外游客7.23万人次，增长22%；旅游经济总收入30.86亿元，增长23%。旅游规划编制加快，旅游门户网站初步建成。老城区旅游工作全面启动，御窑遗址博物馆一期工程全面完工并正式开放，景德镇陶瓷文化博览区被授予“国家文化产业示范基地”，建设陶瓷名人艺术馆3家。农业生态旅游和农家乐旅游加快发展，A级景区创建取得重大进展。旅游行业管理水平不断提升，旅游购物、就餐等各类标识标准化建设不断完善，在全市旅游星级饭店、主要旅游景区设置中、英、日、韩四国语言文字的标示牌，在市区主要道路口安装了旅游交通指示牌。

　　城市建设势头强劲。政府主导投入的工程全面复工，一大批重点工程基本完工。一江两岸美化、亮化、绿化工程全面实施，白鹭大桥顺利竣工，紫晶宾馆投入使用，湖田小区经济适用房主体封顶，西瓜洲污水处理厂主体完工。韭菜园路网、中华北路延伸工程、景德大道、唐英大道、体育中心、西山湖宾馆等重点工程建设不断加快。景鹰高速公路建成通车，湘官一级公路和吴乐秧、景瑶公路加速推进，新建和改造农村公路440公里。电厂“上大压小”、九景衢铁路和德昌高速公路等重大基础设施建设项目前期工作取得新进展。老城区改造稳步推进，戴家弄街区、斗富弄街区、人民公园周边街区和瓷都大道西段改造进展顺利。城市基础设施日趋完善，改扩建城市道路面积19.83万平方米，新建城市绿地面积8.5万平方米，新增城市路灯5000余盏，建成垃圾中转站3座、公厕6座。城市管理长效机制逐步建立，完成25个重点工程审计工作，新建的31条道路由各项目部全面移交建设部门和两区规范管理，在全省率先启动数字化城市管理平台建设。严控违法用地和违章搭建，城区无证私房清理整顿工作扎实开展。土地运作进一步规范，土地经营收益创历史新高，土地净收益4.51亿元，同比增长5倍，有效缓解了财政资金短缺压力。城区饮用水源保护进展顺利，实施“5.31零点行动”，全面关停生活饮用水源一、二级保护区排污口。昌江河道采砂整治取得重大成果，采砂秩序得到规范。城市创建卓有成效，荣获“全国绿化模范城市”和“中国人居环境范例奖”等先进称号。

　　民生工程扎实推进。预计全年新增城镇就业2.31万人，新增转移农村劳动力2.02万人，“4050”人员再就业1094人，零就业家庭就业安置率100%，城镇就业率达到96.7%。城镇职工（含离退休人员）和农民工参加基本养老保险分别为25.98万人和5100人。城镇职工和农民工参加工伤保险分别为16万人和1.8万人。城镇职工基本医疗保险参保20.1万人，困难国有企业职工参加医疗保险3.05万人，城镇居民基本医疗保险参保15万人。城乡低保救助分别为4.98万人和3.34万人。城市困难群众大病救助7499人，农村困难群众大病救助（含农村合作医疗）3.95万人。全面推行新型农村合作医疗制度，参合农民80.67万人，参合率86.8%。完成141个村卫生室（所）、15个乡镇卫生院、12个社区卫生服务机构达标建设。解决农村饮水困难人口2.1万人。义务教育阶段免除学杂费学生18.85万人，义务教育阶段免费提供贫困家庭学生教科书3.2万人。新增享受廉租住房补贴家庭143户。完成公积金归集1.5亿元，增长34%，累计发放个人贷款4.4亿元，解决5674户职工家庭购房贷款难问题。行政村道路硬化率达到90%。农村沼气池新增4000口。

　　社会事业协调发展。科技进步不断加强，共申报部、省科技项目71项，立项36项。获全省科技进步一等奖、二等奖各一项。国家知识产权试点工作深入推进，专利申请量 165件。《景德镇专利资助暂行办法》正式实行，共资助专利15项。昌汽、华意和中景集团跻身首届中国科技名牌500强。教育事业蓬勃发展，素质教育积极推进，顺利通过“两基”国家级检查，新五中开工建设，全市小学适龄儿童入学率达100%，年辍学率0.1%，初中阶段年辍学率2.03%；高中毛入学率提高5个百分点，达到65.2%；中等职业教育完成省扩招计划，普职比达到6：4；各项学科竞赛、民办教育、高等教育、老年教育等成果斐然。文化产业快速发展，预计实现文化总产值1.37亿元。群众文化活动中心投入使用。对外文化交流取得新成果，在“俄罗斯中国文化年”活动中，成功举办“白色的金子”—中国瓷器精品展。文物保护得到加强，公布市级非物质文化遗产保护目录40项。广播电视无线覆盖工程抓紧落实，20户以上已通电自然村广播电视“村村通” 完成200个。公共卫生体系不断健全，农村医疗机构加快建设，城区街道社区卫生服务中心覆盖率达100%。体育事业加快发展，群众体育运动广泛开展。人口计生工作取得新进展。食品药品专项整治取得明显成效。绿色生态瓷都建设有效开展，节能减排年度指标全面完成。社会治安综合治理深入推进，维稳工作机制不断健全，公安“三基”工程和信息化建设取得新成果，治安防控体系建设全面启动，三级监控管理平台和259个公共视频监控点建设全面完成。积极推进国防后备力量建设，扎实开展双拥共建活动，实现全国双拥模范城“四连冠”。市中级人民法院审判综合大楼全面竣工，市检察院技术侦察大楼交付使用。普法工作扎实有效，信访工作进一步加强。财税金融改革加快，成效显著。安全生产监管力度加大，非法煤矿实现“零开采、零死亡”。人事编制、统计、民族宗教、外事侨务、新闻出版、老龄、妇女儿童、残联、人防、档案、地方志等各项工作也都取得了新的进步。

　　行政效能有所改善。认真执行市人大及其常委会的决议、决定，及时办理人大代表意见、建议155件，政协提案283件。注册办证“一站式”审批服务水平不断提高。进一步规范了土地运作、有关费用减免等事项审批程序。加强规范性文件审查工作，完成政府规范性文件、其他文件和协议的法律审查161件。审计监督机制逐步健全，职能进一步完善。经济发展环境不断优化，外商投诉问题集中处理协调机制日益健全，民主评议政风行风和投资环境集中整治活动成效明显。“政风行风热线”工作深入开展，“市长短信”平台建设不断完善，办理成效不断提高，“亲商、富商、安商”氛围日渐浓厚。

　　各位代表，过去的一年，瓷都大地处处涌动着思发展、谋跨越的创业热潮，全市上下心齐、气顺、劲足、和谐，实现了我市经济和社会事业的全面发展。这些成绩的取得，是省委、省政府和市委坚强领导的结果，是市人大、市政协大力支持、有力监督的结果，是全市人民团结奋斗、辛勤劳动的结果，也是与历届政府打下的扎实基础分不开的。在此，我代表市人民政府，向全市广大工人、农民、知识分子、干部和离退休老同志，向各民主党派、工商联、无党派人士和社会团体，向驻市解放军、武警官兵和公安干警，向所有关心、支持瓷都发展的海内外友好人士，表示衷心的感谢，并致以崇高的敬意！

　　在看到成绩的同时，我们也清醒地看到经济社会生活中还存在不少困难与问题。主要是经济总量仍然偏小，总体发展水平还不高；重点领域和关键环节改革难度仍然较大，文化旅游产业的优势还没有得到充分发挥；部分群众生活仍比较困难，影响社会和谐稳定的因素还不少；经济发展环境和行政效率仍有待进一步改善和提高等。对于这些困难和问题，我们将高度重视，并通过发展在今后的工作中努力克服和解决。

　　二、2008年工作任务和主要措施

　　2008年政府工作的总体要求是：全面贯彻党的十七大和中央经济工作会议精神，高举中国特色社会主义伟大旗帜，深入贯彻落实科学发展观，始终坚持以经济建设为中心，坚持解放思想，坚持改革开放，坚持又好又快，按照省委、省政府一系列重大战略部署，在市委的领导下，以项目建设为抓手，突出增加投入、做大总量、减轻包袱、盘活存量，实现“经济重镇、旅游都市、特色瓷都”建设的新跨越。

　　2008年全市国民经济和社会发展的主要目标是：全市生产总值增长13%，财政总收入增长16%，其中地方一般预算收入增长16%；城镇固定资产投资增长20%，社会消费品零售总额增长16%；出口总额增长20%，实际利用外资增长30%，实际利用内资增长40%；城镇居民人均可支配收入增长10%，农民人均纯收入增长8%；城镇登记失业率控制在4.3%以内；单位GDP能耗下降4%，主要污染物排放总量减少3%以上；居民消费品价格指数涨幅控制在4.5%以内；人口自然增长率控制在8‰以内。

　　实现上述目标，要以工业化为核心，以大开放为主战略，以科技创新和机制体制创新为强动力，努力做到“三个必须坚持”和“三个更加注重”，即：必须坚持以增加投入、做大总量为目标，主要经济指标保持两位数以上增长；必须坚持以推进重大项目建设为关键举措，牢固树立抓项目促发展的理念；必须坚持以发挥我市比较优势和后发优势为着力点，积极捕捉机遇、善于抢抓机遇、敢于用好机遇，推进瓷都跨越发展。同时，更加注重挖掘陶瓷历史文化资源，更加注重提高经济发展质量和效益，更加注重生态文明建设，不断增强可持续发展能力。

　　（一）以项目建设为抓手，加快推进新型工业化进程

　　把项目建设放在经济工作的突出位置，大力实施工业强市战略，促进经济集约发展，实现“345”发展计划，即规模以上工业销售收入增长30%；工业项目投资增长40%；规模以上工业利税增长50%。到2010年，实现规模以上工业增加值200亿元的“三年翻番”发展目标。

　　实施一批重大项目。市一级突出抓投资亿元以上工业项目，县（市、区）突出抓投资5000万元以上工业项目。开工建设嘉和公司汽车零配件产业基地宇强机械、长宇机械、新宏声机械、易宏配件等重点机械制造项目，加快乐平龙盛科技园建设。继续推进长虹机电产业基地和江西美菱冰箱生产线、锦溪水泥日产4500吨熟料生产线、富祥药业一期、宏柏化工等一批已开工项目建设进度，争取早日竣工投产。着力抓好焦化干熄焦节能减排、正裕配件和嘉鸿钢结构等一批重点项目达产达标。2008年，工业项目完成投资65亿元。

　　提高工业发展水平。2008年，全市规模以上工业增加值增长25%，达到125亿元；销售收入完成312亿元；利税完成15亿元。扶持一批骨干企业做大做强，力争销售收入超亿元企业达到40户，其中，超30亿元企业3户；利税超千万元企业达到20户；民营工业产值超千万元企业180户。全市规模以上工业增加值占GDP的比重提高4个百分点。提升发展质量，突出抓好焦化、陶瓷股份公司等重点企业技术改造，提高企业管理水平，降低企业成本。2008年，力争全市工业经济效益综合指数提高15个百分点，达到145 %。

　　增强自主创新能力。创新是经济活力的源泉，创新有突破，发展才有裂变。不断加快昌飞、昌汽、华意、中景等骨干企业的技术创新中心建设，促进一批具有自主知识产权的技术转化为现实生产力，搭建技术创新和转化的共享平台。完成重点新产品开发项目10个，完成重点新产品试产项目10个，新增省级以上高新技术企业3至6家，积极组织申报各类科技项目，力争立项40项，争取省科技厅与景德镇市共建省陶瓷产品设计中心。保护“景德镇”、“华意”、“昌河”和“红叶”等知名品牌，注重把品牌优势转化为资源优势、产业优势和经济优势。

　　加快重大基础设施项目建设。开工建设九景衢铁路、景德镇机场改扩建、景德镇港客货运码头复建项目。积极配合做好景德镇绕城高速公路湘湖至丽阳段续建工程。积极争取投资50亿元的电厂“上大压小”项目、投资11.12亿元的浯溪口水利枢纽工程项目、投资90亿元的皖赣铁路复线改造项目立项、开工建设。投资1.5亿元新建改建农村公路373公里。

　　（二）以工业园区建设为主战场，大力发展开放型经济

　　坚定不移地实施以工业化为核心、以大项目为抓手的大开放主战略，充分利用好国际国内两个市场、两种资源，努力扩大对外开放的广度和深度。实现“234”目标，即出口总额增长20%，达到1.92亿美元；利用外资增长30%，达到1亿美元；利用内资增长40%，达到68亿元。

　　打造园区发展平台。支持各种要素向工业园区倾斜，围绕基础设施、招商引资、产业聚集、高效服务四个重点，突出园区发展特色，构建产业配套体系，加快推进工业园区建设。2008年，园区规模以上工业总产值占全市规模以上工业比重达40%以上，主营业务收入110亿元，增加值45亿元，利税9亿元。重点打造高新技术产业园区、陶瓷工业园区和乐平工业园区，加快建设机械电器、陶瓷和精细化工三大产业板块基地。2008年，高新技术产业园区规模以上企业达到70家，实际引进资金50亿元，完成工业总产值60亿元。陶瓷工业园区规模以上企业达到30家，实际引进资金11亿元，完成工业总产值15亿元。乐平工业园区规模以上企业达到60家，实际引进资金22亿元，完成工业总产值75亿元。依托昌飞直升机生产线扩建、天人电子和多晶硅等项目建设，积极引进配套企业，不断延长产业链，加紧培育航空产业、电子信息产业和半导体产业基地。

　　大力开展招商引资。把引进工业项目作为招商重点，确保引进资金占总量的65%以上。围绕我市工业主导产业引进一批配套项目、上下游项目，扩大产业配套和企业集群；注重抢抓国家调整加工贸易政策机遇，做好承接转移的各项工作；把引进外资作为招商重点突破，培育外贸出口企业和人才队伍，保持财政、金融支持外经贸发展优惠政策的连续性，加快发展开放型经济。

　　优化经济发展环境。牢固确立“三分招商，七分安商”理念，进一步规范和完善招商引资优惠政策。建立市级领导带头挂点的重点建设项目和招商项目专人跟进、全程服务制度。积极做好驻市部省属企业服务工作，及时解决企业发展中的实际困难。加快电子政务建设，强化行政服务中心职能，继续发挥外商投诉中心的作用，完善外商投诉制度。加大投资环境专项整治力度，严肃查处侵害投资者权益的违法行为，全力打造景德镇开明开放的新形象。

　　（三）以提高产业竞争力为根本，促进陶瓷产业振兴

　　紧紧抓住国内外陶瓷产业转移、重组的机遇，以省部共建景德镇国家陶瓷科技城为龙头，推进陶瓷产业、科研、人才、交流四大基地建设，实现“5678”发展目标，即陶瓷产值55亿元，陶瓷出口6800万美元，陶瓷固定资产投资7.2亿元，陶瓷产业税收8000万元。

　　打造大陶瓷产业格局。以项目建设促进陶瓷产业发展，加快金意陶、乐华和特地等一批投资10亿元以上的重大陶瓷项目建设进度，积极培育销售规模超5亿元、10亿元的骨干企业群，形成我市陶瓷产业的支撑力量；加快三雄陶瓷、圣泰陶瓷、辰道陶瓷、南瓷绝缘子和西马克电瓷等项目建设，争取早日投产。2008年，新增产值亿元以上陶瓷企业3至5家。推进陶瓷工业园区建设，完善园区水、电、气等配套设施，快速推进园区标准化厂房建设。陶瓷工业园区逐步发展形成以同惠、神飞特陶等为特色的高科技陶瓷生产区；以海畅法蓝瓷、隆祥等为代表的高档艺术瓷、日用瓷生产区；以金意陶、乐华、特地等为重点的建筑卫生陶瓷生产区；以翼龙、协和等项目为依托的相关陶瓷配套生产区。积极支持鼓励陶瓷股份公司、鹏飞建陶等本土企业做大做强，形成内外企业共同发展、国有民营共同促进、各个生产环节协调推进的大陶瓷产业发展格局，力争规模以上陶瓷工业增加值增长100%以上。

　　提高陶瓷创新能力。加快建设国家日用及建筑陶瓷工程技术研究中心，提高陶瓷产业创新能力，争取超耐热陶瓷煲等项目开工建设。充分发挥我市人才、技术优势，加快建立以企业为主体、市场为导向、产学研相结合的技术创新体系，促进陶瓷科技成果向现实生产力转化。注重把陶瓷高新技术的引进消化吸收再创新结合起来，不断推出陶瓷新材质、新器型、新花面、新品种，增添陶瓷发展的生机和活力。加快推行陶瓷标准化建设，进一步加强陶瓷知识产权保护。

　　规范市场经营秩序。按照陶瓷生产园区化、专业化和陶瓷销售规范化、国际化的发展方向，进一步抓好燃气梭式窑的整治和“退城进园”工作。继续抓好陶瓷市场整顿，规范陶瓷市场经营秩序，积极建立长效市场管理机制。抓住“奥运商机”，做好“奥运瓷”的生产、经营工作。扶持重点陶瓷专业市场做大做强，着力将我市培育成集天下名瓷之大成的陶瓷大市场。围绕“一年站稳、三年站好、五年站高”的目标，全力办好2008景德镇国际陶瓷博览会。启动国际陶瓷会展中心建设，提升陶瓷会展水平。

　　（四）以创特色富农民为重点，大力推进社会主义新农村建设

　　围绕“扬优成势重特色，开放发展富农民”的思路，突出加强农业基础建设，大力提高农业综合生产能力，全面落实各项强农惠农政策，千方百计增加财政对农业农村投入，做到财政支农投入的增量明显高于上年，固定资产投资用于农村的增量明显高于上年，政府土地出让收入用于农村建设的增量明显高于上年，积极促进农业稳定发展、农民持续增收，扎实推进新农村建设。

　　加强农业基础设施建设。推进基本农田保护体系、高产稳产农田建设体系、配套服务保障体系建设，加大农田水利基础设施建设力度。落实病险水库除险加固资金投入，完成乐平勤俭、昌江山田等中型水库和50座小型病险水库除险加固工作；开工建设乐平民兵水库、浮梁虎形水库、昌江郭璞峰水库等除险加固项目。继续抓好镇桥联圩、续湖联圩项目建设。推进共库灌区等一批农田灌溉工程建设。认真实施好测土配方施肥和农机购置补贴等一批农业项目。切实控制建设占用耕地及林地，继续大力实施“造地增粮富民”工程，加大土地复垦整理力度，加快中低产田改造，搞好水土保持工作，提高粮食综合生产能力。2008年，全市粮食播种面积稳定在132万亩，力争粮食总产达10.3亿斤。继续抓好退耕还林、长防林和日本政府贷款造林项目，按照“十一五”期末全市森林覆盖率达到65%的目标，2008年完成造林11万亩。

　　突出特色推进农业产业化。继续推进农业产业化“121”工程，即培育壮大1个销售收入超10亿元、2个超亿元、10个超5000万元的龙头企业，建成20个规模相对集中的生产基地，形成100个“一村一品”的特色村。坚持以基地开发促进产业开发，以龙头企业培育带动特色产业规模发展，着力培育特色农业基地。鼓励农业产业化企业利用优质资产、优势产品、优良品牌等对外招商，新增国家级、省级龙头企业4至5家。继续推进农业经济结构战略性调整，抓好乐平蔬菜、浮梁茶叶和昌江养殖三大特色产业带建设，全市养殖业占农业总产值比重提高1个百分点，高效经济作物占种植业比重提高1.8个百分点。发展农民专业合作经济组织，重点建好10个市级和32个县级示范专业合作社。加快建设市现代农业园区、乐平农业科技园，引进和培育特色农产品开发加工企业和高效精致农业项目，做大做强农产品精深加工，带动全市现代农业发展。

　　加快建设社会主义新农村。按照“生产发展、生活宽裕、乡风文明、村容整洁、管理民主”的要求，把发展经济、富裕农民作为新农村建设的首要任务，坚持抓重点、攻难点、出亮点，着力建设一批具有较高标准的示范点。加大农村公共设施建设力度，提高农村公共服务能力。实施好农民培训阳光工程，发挥各类培训基地作用，培训农民工5万人次。加快农村社会事业发展，加大农村教育、文化、卫生、计生等公益事业的投入，结合农村信息化推进工作，建设农村综合文化站、活动中心等，丰富农民精神文化生活，让更多农民群众“走平坦路、喝干净水、上卫生厕、住整洁房、用洁净能源、过文明生活”。

　　大力发展县域经济。紧紧围绕促进农民增收、增加财政收入、壮大经济实力，把县域经济摆到全市经济发展大局中谋划，充分发挥县（市、区）各自比较优势，加快产业聚集和区域特色经济发展。乐平市依托较好的工业基础，以工业为突破口，全力推进工业化进程，力争总量在全省争先进位。浮梁县在建好三龙陶瓷工业基地的同时，注重发展名优特农产品，建设特色农产品市场，大力推进特色农业产业化；要依托良好的生态资源，加强生态资源保护，提高森林覆盖率，加快发展乡村旅游。昌江区要利用城乡结合部的优势，大力发展民营经济，积极参与城市化和大中型企业的协作分工，配套城市生产、生活。珠山区应主动服务城市，加大社区建设力度，大力发展旅游、文化、服务等第三产业。

　　（五）以陶瓷历史文化为依托，提升文化旅游产业发展水平

　　充分挖掘我市厚重的陶瓷历史文化底蕴，打好文化牌，做足瓷文章，以开发老城区旅游为重点，促进陶瓷文化资源优势向旅游产业优势转化。力争全年接待国内游客860万人次，增长13%，境外游客8万人次以上，增长20%；实现旅游经济总收入38亿元，增长25%。

　　精心打造重点景区景点。按照建设国家AAAAA级景区的要求，加强陶瓷文化博览区的功能建设，加快高岭·瑶里景区和浮梁古县衙的基础设施建设，打造景德镇王牌景区。进一步加大德宇龙山生态园、洪岩仙境、杨湾风景区、世界陶瓷公园、金竹山寨风景区、中国瓷园等景区建设力度，争取新建成一批国家AAAA级景区和国家AAA级景区。开发老城区旅游，突出抓好御窑遗址保护工程、祥集弄民居、吊脚楼、三闾庙古码头和刘家弄民窑遗址公园等老城区旅游项目建设，推出1至2条以老瓷行、老坯房、老里弄、古民居等为特色的“老景德镇”精品旅游线路。

　　发展文化创意产业。建设陶瓷文化产业基地和区域性陶瓷文化产业群，培育陶瓷文化产业骨干企业，做大陶瓷文化产业项目，争取环球哈哈泥、海畅法蓝瓷和中国瓷园成功申报全国文化产业示范基地。加强世界陶瓷人才、技艺和作品的交流，完善三宝陶艺村、高岭和雕塑瓷厂等平台建设，新建一批名人陶瓷艺术馆。创新宣传推介形式，拍一部电影或电视剧，创作一首好歌曲，推出一批瓷歌、瓷舞等文艺表演节目，扩大瓷都的影响力。加强非物质文化遗产保护，积极传承民间老艺人陶瓷技艺，提升我市文化旅游特色品牌。

　　积极开拓旅游市场。启动景德镇游客服务中心建设。加强区域联动，注重与婺源、庐山、黄山等周边地区旅游资源的优势互补，努力构建“区域联动、资源共享、优势互补”的联合旅游促销体系。加大旅游宣传推介力度，强化旅游营销手段，提升我市旅游知名度。创新发展机制，整合旅游资源，大力支持旅游企业集团化发展和网络化经营，引进有实力的旅游集团参与我市旅游产业的开发、经营。加快旅游企业所有权、管理权、经营权分离，增强企业发展活力和发展后劲。

　　（六）以国企改革为突破口，加快体制机制创新

　　机制不新难以创新，体制不活难以搞活。要以机制体制创新为强动力，全面推进机制体制改革，增强经济发展的内在动力和活力。

　　深入推进国有企业改革。以建立健全现代企业制度为方向，以公司制、股份制改造为目标，做大做强国有企业，加快中小国有企业改革。大力推进股权多元化，完成华意压缩机股份有限公司战略重组，2008年形成年产能力冰箱60万台、压缩机1800万台，成为国内压缩机行业龙头企业；加快实施焦化煤气总厂、德宇集团、陶瓷股份公司、医药公司等企业改制；依法推进宇宙瓷厂等企业实施政策性破产。继续做好不良资产整体打包回购处置工作，创新不良资产回购处置的有效途径和方法，提高资产利用率和变现率。加大国有资产监管力度，建立健全国有资产监管体系，建立中介机构委托制度，规范企业国有资产评估行为，提高国有资产评估监督管理水平和效率。规范国有产权管理，全面开展产权登记工作，推进市属国有企业的清产核资工作，建立健全现代产权制度。抓好行政事业单位经营性资产统一管理，加强已改制国有企业剩余资产、核销资产、不良债权等资产的管理工作。

　　大力支持金融改革。优化金融环境，建立健全政银企联系的长效机制，完善信用体系，防范金融风险。大力整合和发展地方中小金融机构，推进城市商业银行组建；积极引进国内较大规模的金融企业在我市设立分支机构，推动银行业、证券业、保险业的发展。建立奖励激励机制，充分调动金融机构支持地方经济发展的积极性。通过吸引社会资本参与等方式，进一步加强我市融资担保平台建设。积极拓宽融资渠道，通过公司上市、企业债券、期货等方式提高直接融资比重，做好德宇集团等企业上市准备工作，争取有1至2家股份公司进入上市辅导期。

　　积极推进财税体制改革。继续深化部门预算和国库集中支付制度改革，合理定额经费标准，保障财政资金安全。加快建立健全公共财政体制，新增财力进一步向困难群众、农村、基层和社会事业倾斜。加强财政大额专项资金绩效评估。精心组织和逐步实施好“乡财县代管”改革，加快实行“村帐乡代理”试点。

　　扎实推进农村各项改革。继续深化农村综合配套改革，抓好乡镇职能转变和人员分流，完善教育经费保障机制，进一步推进县乡财政管理体制改革和畜牧兽医管理体制改革。巩固和深化林权制度改革，加快推进林权交易中心建设等配套改革和国有农场、林场改革，建立生态公益林保护机制，实行生态公益林补偿资金“一卡通”。完善农村土地制度改革，加快建立县、乡、村土地台账，形成更加有效的土地保护机制。完善基层农业技术服务体系，强化服务功能。

　　全力推进全民创业。积极推进“十百千”工程，即扶持10个龙头企业、100家民营企业和1000户愿意创业的城市居民和农村农户。实施中小企业和个私企业“成长工程”，不断培育一批扎根本土、具有自主品牌、核心技术和市场竞争力的本土企业，努力建设一支敢领时代潮流、能承担社会责任的企业家队伍。加大创业扶持力度，全面激活创业主体，鼓励和支持经营管理者和科技人员领头创业，吸引外出务工人员返乡创业，扶持下岗失业人员自主创业，推动农村基层干部和群众致富创业。

　　（七）以创建“国家五城”为目标，打造山水生态文化特色城市

　　坚持千年古韵与现代气息相结合，按照“城市向西扩展、工业向南聚集”的总体布局，高起点规划、高标准建设、高效能管理、高水平经营，打造城在山中、水在城中、人在画中的山水、生态、文化特色城市。

　　完善城市规划。城市之美，源于规划。坚持规划优先，突出规划的科学性、前瞻性和严肃性，严格执行规划评审、重大项目专家论证、重点地段建设审批及公示、项目批后跟踪管理和规划执法巡查等制度，坚决做到违反规划的项目不批、不做详规的项目不批、选址不合理的项目不批。积极策应环鄱阳湖城市群打造，加快我市城市发展战略规划。全面启动城市主体功能区规划编制工作，加大推进《景德镇市城市总体规划（2007—2025）》修编和《景德镇市城镇体系规划》、《景德镇市中心城区近期建设规划》、《景德镇市历史文化名城保护规划》等专项规划的编制工作。

　　推进城市建设。重点推进一江两岸改造工程，加快两岸道路及景观带建设，将其打造成我市集防洪、旅游、休闲、文化于一体的核心景观带。完善城市基础设施、功能设施，加快老城区道路改造、修缮，推进景德大道和戴家弄街区、斗富弄街区、人民公园周边街区改造等重点工程建设。实现白鹭大桥全线贯通，完成中国陶瓷博物馆精装修、中华北路延伸工程和体育中心等项目建设，启动中国民居园建设。坚持历史文化遗存和环境风貌保护并重的原则，注重历史文化遗址保护，实施御窑和老汽车站等历史文化遗址保护和修缮改造工作。建设好经公桥镇、鲇鱼山镇等中心镇，全市城镇化率达到53%。

　　搞活城市经营。按照“管住总量、严控增量、盘活存量、节约集约”的原则，建立完善节约集约用地的标准体系，充分利用闲置土地，提高土地利用效率，保障必要的建设用地。全面落实《招标拍卖挂牌出让国有建设用地使用权规定》，经营性建设用地和工业用地全部实行公开招标拍卖挂牌，着力规范土地交易二、三级市场，实现国有土地资产价值最大化。2008年计划控制出让经营性土地1500亩以内，力争土地净收益达5亿元。进一步开放城镇基础设施、市政公用行业市场，坚持“谁投资、谁经营，谁受益、谁承担风险”的原则，吸引国内外投资者参与城市基础设施建设与经营。做好城市无形资产经营工作，充分发挥城市无形资产的经济效益。

　　强化城市管理。把创建作为提升城市整体形象及竞争力的抓手，以创建“国家卫生城市”、“全国文明城市”、“国家环保模范城市”、“国家生态园林城市”、“中国最佳旅游城市”为目标，搞好城市环境综合整治，大力开展违法建设专项整治，加强“城中村”的环境治理，加大对非法用地、乱建私房行为的清理整顿。健全城市管理规章制度，积极推行城市管理综合执法，不断完善数字化城市管理。

　　（八）以民生工程为着力点，统筹推进社会各项事业发展

　　在加快发展中统筹兼顾不同社会群体的利益，统筹发展社会各项事业，注重社会公平，切实解决好群众最关心、最直接、最现实的利益问题，促进和谐社会建设，使发展成果让全市人民共享。

　　积极实施民生工程。就业是民生之本。要以创业带动就业，城镇新增就业人员2.4万人，“4050”人员再就业1300人；新增转移农村劳动力2.1万人；零就业家庭就业安置率100%；跨地区劳务输出20万人。进一步完善社会保障体系，城镇职工（含离退休人员）和农民工参加基本养老保险分别达27.8万人和8000人；城镇企业退休职工养老金标准人均提高100元；城镇职工基本医疗保险参保23.2万人；国有困难企业职工参加医疗保险5.5万人；城镇职工工伤保险参保18万人，农民工参加工伤保险3万人；城镇居民全部纳入基本医疗保险范围；扩大农村低保范围，适当提高财政补差标准；提高农村“五保户”集中供养水平，集中供养率达到80.8%；将新增农村低保对象全部纳入农村医疗救助范围；创建食品药品安全示范县，深入推进农村药品“两网”建设，建立覆盖城乡居民的食品药品安全保障体系；全面完成教育民生计划；以“菜篮子”为重点，抓好生活必需品供应，确保数量充足、价格平稳、秩序井然。

　　扎实为民办好十件实事：（1）加快推进市第五中学、梨树园小学、市第二小学和农村初中改造等教育设施建设，改善基础教育办学条件。（2）完成市妇幼保健院新病房大楼建设，改善妇女儿童就医条件；建设一座容纳500观众的小剧场，完成光明电影院改造工程，解决市民看戏看电影难的问题。（3）完成西瓜洲污水处理厂工程，启动高新区污水处理厂建设，新建2个停车场，2个洗车场，2座垃圾中转站、20座公厕，改造20座旱厕，添置2000个果壳箱，提高市政公用设施水平。（4）完成一江两岸城区段景观建设工程，新建5个市民休闲亭，实施沿河东西路、中山路、中华路、解放路和30条里弄改造，加强城市主干道各支线道路硬化建设，完成湖田大桥扩建工程，改善老城区居住环境，解决群众出行难问题。（5）新建10万平方米经济适用住房；新建100套廉租住房，廉租补贴受益1100户，解决中低收入家庭住房难问题。（6）全面完成20户以上已通电自然村广播电视“村村通”建设任务。（7）开工建设500千伏景德镇输变电工程，完成220千伏三龙输变电工程和110千伏马鞍山输变电工程，提高工业和城市生活用电质量。（8）实施城乡饮水安全工程，解决城市居民和2.3万农村人口饮水难问题。（9）提高农村最低保障制度覆盖率，农村低保在现有基础上扩面50%，并适当提高补助标准，解决农村低收入家庭生活困难问题。（10）改善农村交通条件，新建鲇鱼山、鹅湖、三龙等7个农村客运区乡站和60个村庄候车亭。

　　统筹发展科技教育等各项社会事业。实施人才强市战略，加强人才队伍建设，培养、吸引和用好创新型专业人才和高技能人才。以加强企业自主创新，发展高新技术产业为重点，增强自主创新能力，加快科技成果产业化，争取获省以上科技进步奖2个，列入省科技成果产业化项目2个，高技术产业增加值占GDP达到8.6%。加快民营科技企业发展，争取新认定民营科技企业8家。落实优先发展教育战略，推进素质教育，注重教育公平，优化教育结构；促进义务教育均衡发展，加快普及高中阶段教育，大力发展职业教育，提高高等教育质量，重视学前教育，关心特殊教育，积极发展民办教育，加强老年教育网络建设，使各级各类教育协调发展。促进文化艺术事业的大发展大繁荣，积极开展群众性文体活动，培育富有地方特色文化，打造一批文化精品；开展第三次文物普查工作，加强文物和非物质文化遗产保护。不断改进医疗卫生服务，全面完成市传染病医院国债建设项目，完成“120”急救中心组建并投入运行，积极争取全国地市级中医院建设项目并抓紧实施。广泛开展全民健身活动，提高群众身体素质和体育竞技水平。进一步加强国防教育和国防后备力量建设，不断推进双拥共建活动深入开展。

　　注重经济社会可持续发展。坚持走生产发展、生活富裕、生态良好的文明发展道路，建设资源节约型和环境友好型社会，推进绿色生态瓷都建设。积极推进资源综合利用，大力发展循环经济，推进清洁生产，加强环境保护，努力实现节约发展、清洁发展、安全发展和可持续发展。大力实施节能减排重点工程，抓好重点耗能行业余热余压利用、能量系统优化以及工业锅炉（窑炉）改造等10个节能项目，推进黑猫炭黑、景德镇电厂、江西电化厂和江西化纤化工公司生产废水循环利用等项目，关停城市建成区燃煤小锅炉等项目，同时对消耗高、污染重、技术落后的工艺和产品实施强制性淘汰制度。推进生态瓷都“十大工程”建设，搞好“七个专项整治”。坚持生态涵养与环境治理并重，加强生态体系建设，重点保护好森林资源。加强土地管理，合理开发土地资源，提高土地集约节约利用水平。发展壮大矿业经济，坚持整顿秩序与整合资源并重，实行规模办矿、集约办矿，努力提高矿业的集约化程度。继续加强人口计划生育工作，稳定低生育水平，提高出生人口素质，综合治理出生人口性别比失衡。

　　全力维护社会和谐稳定。提高社会管理水平，加强“和谐平安瓷都”建设。正确处理好人民内部矛盾，尤其要处理好征地、拆迁、企业改制等涉及群众切身利益的问题，维护群众的合法权益。加强和改进信访工作，坚持“谁主管、谁负责”的原则，落实“一把手”负责制，加大信访案件督查督办力度，强化矛盾纠纷排查和超前化解工作，依法及时合理地处理群众反映的突出问题，把矛盾和纠纷解决在当地、解决在基层、解决在萌芽状态。推进社会治安综合治理，进一步完善维护稳定工作机制和社会治安防控体系，加大严打整治力度，杜绝重大群体事件发生，切实增强公众安全感，保障人民群众安居乐业。完善突发事件应急管理机制，建立健全社会预警体系，加大社会利益协调和社会纠纷调处力度，提高防范和处置突发事件的能力。深化普法宣传工作，增强公民法律意识。高度重视安全生产工作，强化责任制，加强督促检查，建立健全安全生产长效机制和应急救援机制，加大事故责任追究和处罚力度，杜绝重特大事故发生，确保人民群众生命财产安全。

　　（九）以行政能力建设为切入点，加强政府自身建设

　　坚持科学执政、民主执政、依法执政，努力建设“和谐、创新、务实、高效、自律”的政府。

　　坚持求真务实，加强能力建设。牢固树立正确的政绩观，把抓发展、抓项目建设作为提高行政能力的重点，不断提高各级政府特别是各级领导驾驭市场经济、领导经济发展的能力，增强干部的理解力、执行力、操作力。牢固树立“工作以经济建设为中心，经济建设以项目为核心，不抓项目就是失职，不会抓项目就是不称职”的理念。围绕发展抓项目，把握国家政策导向，熟悉项目论证、立项、申报、实施等程序，增强资本运作本领，把每个干部都培养成为抓项目的行家里手。解放思想，勇于创新，大胆实践，努力做到决策有新思维，工作有新思路，破解难题有新举措，说实话、办实事、求实效。

　　坚持依法行政，打造诚信政府。全面实施依法行政纲要，加强和改进行政执法工作，切实做到严格执法、公正执法和文明执法。强化行政执法监督，严格实行执法责任制和执法过错追究制，切实做到有权必有责、用权受监督、侵权须赔偿、违法要追究。健全公平、公开、公正的行政执法评议考核机制。完善各级政府诚信考核和监督执政承诺的制度建设，敢于面对、解决历史遗留和现实存在的问题，保证行政行为的透明性和连续性；重信用、守合同，以改善环境的实际成效取信投资者，以明晰的责任体系塑造政府诚信形象。

　　坚持民主决策，实行政务公开。进一步完善重大决策的规则和程序，推进依法决策、科学决策和民主决策。自觉接受人大及其常委会的法律监督和政协的民主监督，认真听取各民主党派、工商联、无党派人士和人民团体的意见，接受新闻舆论和社会公众监督。坚持市长手机短信号码公开办理制度，畅通“12345”为民服务热线。按照 “公开、透明、便于监督”的原则，增强权力运作透明度，做到阳光审批、阳光收费、阳光处罚、阳光交易，使社会普遍关注的热点问题始终处于人民群众的监督之下。

　　加强政风建设，严格廉洁自律。牢固树立机遇意识、忧患意识、责任意识、效率意识，始终保持奋发有为、昂扬向上的精神状态。深入基层、深入实际、深入群众调查研究，及时解决基层和群众关心的热点难点问题。强化决策督查，保证工作落实。围绕省政府经济社会考核评价体系，完善目标责任管理考核制度，严肃处理行政不作为和乱作为行为。加强审计和监察工作，建立科学的监督制约机制，做好领导干部经济责任审计和离任审计，规范建设工程招投标、土地使用权转让、政府集中采购和会计集中核算的运作。认真开展商业贿赂专项治理工作，以完善惩治和预防腐败体系为重点，加强反腐倡廉工作。加大大案要案查处力度，以卓有成效的反腐倡廉成果取信于民。不断增强公务员队伍自律意识，廉洁从政，勤政为民，做到自重、自醒、自警、自励。

　　各位代表，今年是贯彻落实党的十七大精神第一年，是实施“十一五”规划承上启下的关键一年，让我们高举中国特色社会主义伟大旗帜，紧密团结在以胡锦涛同志为总书记的党中央周围，全面落实科学发展观，在省委、省政府和市委的领导下，聚精会神搞建设，一心一意谋发展，众志成城求跨越，与时俱进，团结拼搏，扎实工作，为实现经济重镇旅游都市特色瓷都建设的新跨越作出更大贡献！
